# Scanning Tunneling Microscopy for Molecules: Effects of Electron Propagation into Vacuum

**DOI:** 10.1021/acsnano.3c12315

**Published:** 2024-04-29

**Authors:** Abhishek Grewal, Christopher C. Leon, Klaus Kuhnke, Klaus Kern, Olle Gunnarsson

**Affiliations:** † Max-Planck-Institut für Festkörperforschung, Heisenbergstraße 1, Stuttgart 70569, Germany; ‡ Institut de Physique, École Polytechnique Fédérale de Lausanne, Lausanne 1015, Switzerland

**Keywords:** scanning tunneling microscopy, electronic transport gap, single molecule imaging, decoupling layer, thin insulator, phthalocyanine, NaCl

## Abstract

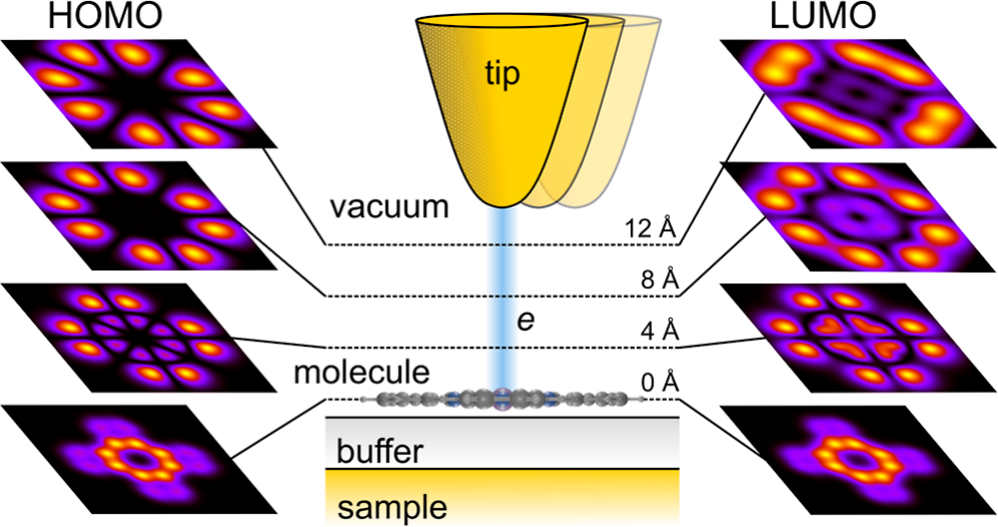

Using scanning tunneling microscopy (STM), we experimentally and theoretically investigate isolated platinum phthalocyanine (PtPc) molecules adsorbed on an atomically thin NaCl(100) film vapor deposited on Au(111). We obtain good agreement between theory and constant-height STM topography. We theoretically examine why strong distortions of STM images occur as a function of distance between the molecule and the STM tip. The images of the highest occupied molecular orbital (HOMO) and the lowest unoccupied molecular orbital (LUMO) exhibit for increasing distance, significant radial expansion due to electron propagation in the vacuum. Additionally, the imaged angular dependence is substantially distorted. The LUMO image has substantial intensity along the molecular diagonals where PtPc has no atoms. In the electronic transport gap, the image differs drastically from HOMO and LUMO even at energies very close to these orbitals. As the tunneling becomes increasingly off-resonant, the eight angular lobes of the HOMO or of the degenerate LUMOs diminish and reveal four lobes with maxima along the molecular axes, where both, HOMO and LUMO have little or no weight. These images are strongly influenced by low-lying PtPc orbitals that have simple angular structures.

Scanning tunneling microscopy (STM) was developed across several papers by Binnig et al.^1−4^ The technique has been extensively discussed in reviews,^5−9^ as well as in early theoretical works.^10−25^ Significant theoretical progress was achieved by Tersoff and Hamann.^10,11^ They assumed that the electrons tunnel to or from an s-orbital on the tip. Using the Bardeen theory,^26^ they demonstrated that the tunneling current is determined by the hypothetical value of the wave function for the tunneling electron at the center of the s-orbital. Substantial effort has been dedicated to improving this assumption.^13,17−25,27−32^ However, these refinements necessitate a thorough understanding of the electronic structure of the tip. Given the limited information available, we adopt the assumption of Tersoff and Hamann. Repp et al.^33^ introduced a NaCl buffer when studying a molecule to reduce the influence of the substrate. They observed that STM shows spatially expanded images of molecular orbitals (MOs) of a pentacene molecule.^33^ Ab initio calculations of STM images have been performed for clean surfaces and for very small molecules adsorbed directly on metal surfaces.^15,16,34−42^ More closely related to the present work are studies of single copper phthalocyanine (CuPc) molecules using a model that includes the highest occupied MO (HOMO), the lowest unoccupied MO (LUMO), and one σ-orbital, located just below the energy of the CuPc HOMO.^43−45^ CuPc has been widely studied due to its demonstration of negative differential resistance,^46^ while the related compound H_2_Pc has garnered significant interest due to observations of up-conversion electroluminescence at tunneling energies in the H_2_Pc transport gap.^47−49^ These experiments raise important and fundamental questions about electrons tunneling through the transport gap.

Here, we study a similar organic luminescent system, namely, PtPc adsorbed on a few layers of a NaCl(100) film on an Au substrate, as illustrated in Figure 1. The molecule is adsorbed with its center metal atom Pt atop an Na site with its isoindole “arm” aligned to the NaCl(100) direction.^49,50^ In particular our study serves as a means to better understand the very nature of tunneling through vacuum in STM studies. We show how images of the HOMO and the LUMO substantially expand radially and explain their origin. The LUMO image also distorts angularly and has significant weight even at spatial positions where the underlying molecule has no atoms for reasons to be discussed below. The images of electrons tunneling through the transport gap are shown to differ drastically from the HOMO or LUMO images, even for energies very close to these states. We show that in an expansion in terms of MOs this is due to contributions from MOs with few nodal surfaces and at substantially lower energy than the HOMO. This effect is very important for understanding experiments manipulating electrons (tunneling through the gap) and photons.

**Figure 1:**
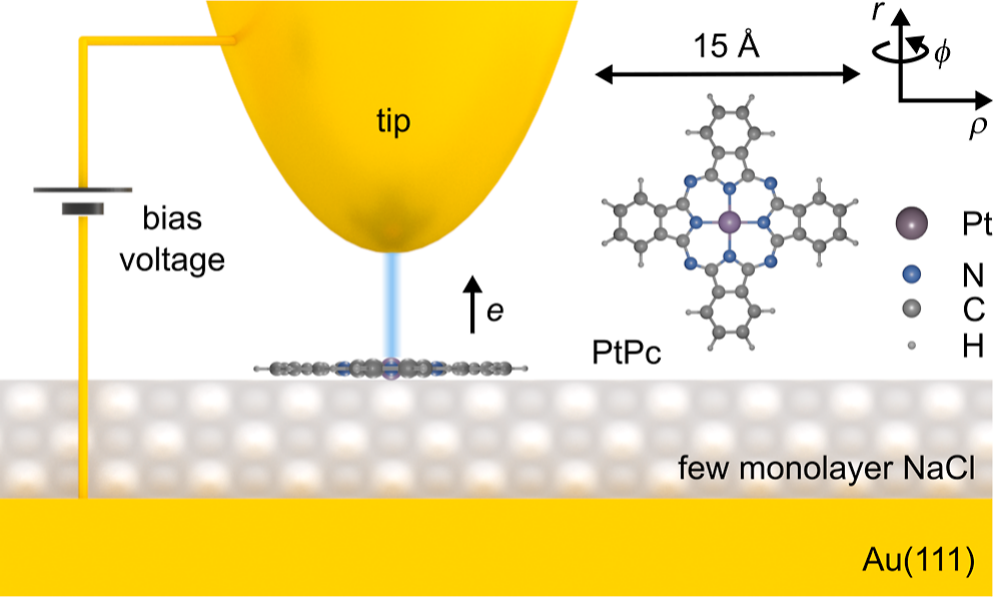
Scheme of the system investigated experimentally and simulated theoretically: PtPc on a thin NaCl(100) film on Au(111) in the tunnel junction of an STM. The four “arms” of the molecule are the isoindole units, shown aligned with the cardinal directions. The origin of the coordinate system is located at the Pt atom and ϕ = 0 is along one of the isoindole “arms”.

Here, we treat a single PtPc molecule adsorbed flat on the surface. We use cylindrical coordinates in vacuum and introduce basis states. In planes parallel to the surface, we use trigonometric functions, cos(*m*ϕ) and sin(*m*ϕ), to describe the angular behavior, and integer Bessel functions, *J*_*m*_(*k*_*mi*_ρ) with *i* – 1 radial nodes, to describe the radial behavior. Perpendicular to the surface, the basis states are described by exponentially decaying functions. For the Au–NaCl–PtPc system, we employ a tight-binding (TB) formalism.^50,51^

We use basis states, |α⟩, in vacuum outside the sample with a given kinetic energy, *T*, determined by the bias. *T* contains positive contributions from the angular and radial variations of the basis state parallel to the surface, *T*_α_^∥^, and negative contributions from the exponentially decaying part perpendicular to the surface, *T*_α_^⊥^
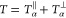
1

We study tunneling electrons with energies ranging from the HOMO to the LUMO, including energies in the transport gap.

Basis states with (a) radial functions with many nodes (large *k*_*mi*_) or (b) angular functions with many nodes (large *m*) have large (positive) contribution, *T*_*mi*_^∥^, to the kinetic energy due to many nodes in planes parallel to the surface. These functions are combined with rapidly decaying exponential functions perpendicular to the surface, which have large negative contributions, *T*^⊥^, to the kinetic energy. This way we obtain basis states with a total kinetic energy, *T*, corresponding to the bias. Such states decay rapidly in the perpendicular direction and play a small role for the topography image at the tip. This effect exponentially favors components with (a) small *k*_*mi*_ and (b) small *m*, i.e., components with few nodes in the angular and radial parts of the wave function.

Factor (a) results in a significant expansion of the STM image in the radial direction when the smallest significant value of *m* is not too small. For PtPc, this phenomenon is applicable to both the HOMO and LUMO. To observe the effects of factor (b), we notice that PtPc has four “arms” along the *x*- and *y*-axis. The HOMO exhibits a total of eight lobes, with a pair of lobes tending to align along each of the molecular arms. Due to factor (b), this tendency diminishes during electron propagation into vacuum. The 2-fold degenerate LUMOs collectively form a set of eight lobes. Due to factor (b), its image markedly accumulates intensity between the molecular arms in the *x* ± *y*-directions, even though there are no atoms in the underlying molecule in these directions. While factor (b) tends to position the eight lobes of the HOMO at equal angles, the LUMO has a tendency to overshoot.

Propagation through the transport gap becomes important for electron and photon manipulations. Factors (a) and (b) mentioned above exponentially favor contributions from basis states with few angular and radial nodes. In ref (50) we emphasized how propagation through the Au–NaCl–PtPc system also favors this type of basis states. Consequently, even for energies very close to the HOMO or LUMO, the in-gap image differs qualitatively from the HOMO and LUMO images. While the HOMO, as well as the two degenerate LUMOs together, have eight lobes, the in-gap image has only four lobes. Despite the HOMO having no intensity right on the *x*- or *y*-axes, and the LUMO having little intensity as well, the image in the gap has its four lobes centered on these axes. Specifically, in an expansion in terms of MOs, we find that the lowest π orbital, with no nodes in planes parallel to the surface, has a large weight due to its small *T*^∥^. More detailed information about the model can be found in the Supporting Information. The next section provides a detailed description of the Theoretical Formalism. In the following section, we present the Experimental Section and Theoretical Results.

## Results and Discussion

### Theoretical Formalism

We study a PtPc molecule adsorbed on a NaCl(100) film of three atomic layers on an Au(111) substrate. As in earlier publications,^50,51^ we use a TB formalism to describe the Au–NaCl–PtPc system, essentially following prescriptions of Harrison (see the Supporting Information).^52^ The resulting Hamiltonian is
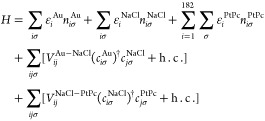
2

The first three terms encode the energies of the Au states, the NaCl states, and the PtPc states, respectively. The last two terms encode the coupling between Au and NaCl, and between NaCl and PtPc, respectively. This Hamiltonian is used for describing the Au–NaCl–PtPc system inside a matching plane at *z*_0_ = 1 Å outside the nuclei of the PtPc molecule. The calculations account for the primarily Cl character of the states in the NaCl band gap due to the mainly Cl character of the NaCl valence and conduction band.^51,53−55^

Beyond the matching plane, we introduce cylindrical coordinates with the radial coordinate, ρ, the azimuthal angle, ϕ, and a coordinate perpendicular to the surface, *z*. The origin of the coordinates is assumed on the center of the molecule (the Pt atom) and ϕ = 0 is on the symmetry line along an isoindole “arm”. We follow Tersoff and Hamann^10,11^ and assume that the important orbital on the tip is an s orbital. The tip has a local radius of curvature *R*. The center of the curvature is located a distance *z* from the surface, which is also the center of the tip s orbital. The tip apex is then at a distance *z* – *R*. In the following, we do not specify the value of *R*, and present our results as a function of *z*, corresponding to a tip distance of *z* – *R*. Furthermore, we neglect changes of the potential in the NaCl–PtPc-vacuum system induced by the tip.

Under these assumptions, the tip does not explicitly factor into the calculations. Coulomb interactions are also not explicitly taken into account. However, the HOMO and LUMO positions are adjusted to the measured values determined from scanning tunneling spectroscopy (STS). As long as the important PtPc states are the neutral ground-state and states with one extra electron or one hole, Coulomb effects and image potential effects are implicitly accounted for by using level positions determined by STS. This formalism, however, is incapable of describing exciton effects. The TB solutions for substrate-barrier-molecule complex are matched continuously to the vacuum solution outlined below.

For *z* > *z*_0_, we assume that the potential *V*(ρ, ϕ, *z*) takes the work function value *V*_0_ = 4.3 eV^56^ inside a cylinder radius ρ_0_ = 12 Å, and positive infinity outside. Then, the potential *V*(ρ, ϕ, *z*) can be expressed as follows
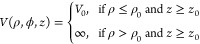
3

The energy zero is set at the Fermi energy of the substrate. The cylinder radius (12 Å) significantly exceeds the distance from the cylinder axis to the outermost H atoms (7.6 Å) or the outermost C atoms (6.6 Å). Consequently, it is then a reasonable assumption that the true wave function of the tunneling electrons is localized within the cylinder. We introduce the Schrödinger equation for an electron with the energy ε(<*V*_0_)
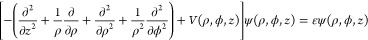
4

Here, we have expressed energies in Rydberg units (Ryd = 13.6 eV) and lengths are given in Bohr radii (*a*_0_ = 0.529 Å). A solution in vacuum outside the PtPc molecule can be written as follows
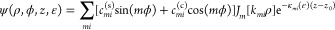
5where *m* is a non-negative integer, and *J*_*m*_ is an integer Bessel function. The coefficients *k*_*mi*_ are defined such that *J*_*m*_[*k*_*mi*_ρ_0_] = 0, ensuring that the wave function is zero for ρ = ρ_0_. To obtain the correct energy ε, we require
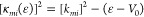
6

For *m* ≥ 1, sin(*m*ϕ) and for *m* ≥ 0, cos(*m*ϕ) describe the azimuthal angle ϕ dependence, while the Bessel functions *J*_*m*_[*k*_*mi*_ρ], *i* = 1, 2, ... describe the radial behavior for a given *m* value.

The factor exp[−2κ_*mi*_(ε)*z*] describes the exponential decay of the square of the wave function in the *z*-direction. This factor introduces a strong energy dependence via the energy dependence of κ_*mi*_(ε) in eq 6. The leading contribution results from exp[−2κ_01_(ε)(*z* – *z*_0_)]. This factor varies over 2 orders of magnitude as the energy ranges from the bottom of the electronic transport gap, at −1.3 eV, to the top, at 1.7 eV. Additional relative variations among the different components are induced by exp{−2[κ_*mi*_(ε) – κ_01_(ε)](*z* – *z*_0_)}, as discussed in Table 1 below. These considerations are rooted in the assumption that the potential reaches its vacuum value directly outside the molecule. In this approach, we have neglected the potential from the tip. This potential is substantially higher in the study of the LUMO compared to that for the HOMO. If this potential were included in the calculation, the pronounced enhancement of the LUMO versus the HOMO would be substantially smaller. Furthermore, the potential is lowered outside the molecule, which we have neglected. The image potential also contributes beyond what is implicitly included in the HOMO and LUMO positions. While these factors should alleviate the strong energy dependence, they are neglected here. These dependencies exist alongside the effects that occur during the propagation through the NaCl buffer and the PtPc molecule. The images are constructed by introducing an artificial Gaussian broadening of 0.05 eV full width half-maximum so that a finite number of states contribute to the image.

**Table 1:** κ_*mi*_(ε) (Å^–1^) Determining the Decay of the *z*-Dependent Functions [eq 6]^a^

*i*	*m* = 0	*m* = 1	*m* = 2	*m* = 4	*m* = 8
1	1.08 (1.000)	1.11 (0.637)	1.15 (0.358)	1.24 (0.084)	1.47 (0.002)
2	1.16 (0.294)	1.21 (0.122)	1.27 (0.046)	1.41 (0.005)	1.71 (0.000)
3	1.28 (0.039)	1.36 (0.012)	1.44 (0.003)	1.60 (0.000)	1.95 (0.000)
4	1.45 (0.003)	1.54 (0.001)	1.63 (0.000)	1.81 (0.000)	2.19 (0.000)

a The numbers in parentheses show the relative damping exp{−2[κ_*mi*_(ε) – κ_01_(ε)](*z* – *z*_0_)} of the intensity of a component relative to the *m* = 0 and *i* = 1 component for *z* – *z*_0_ = 8 Å and ε = 0.

### STM Measurements

**Figure 2:**
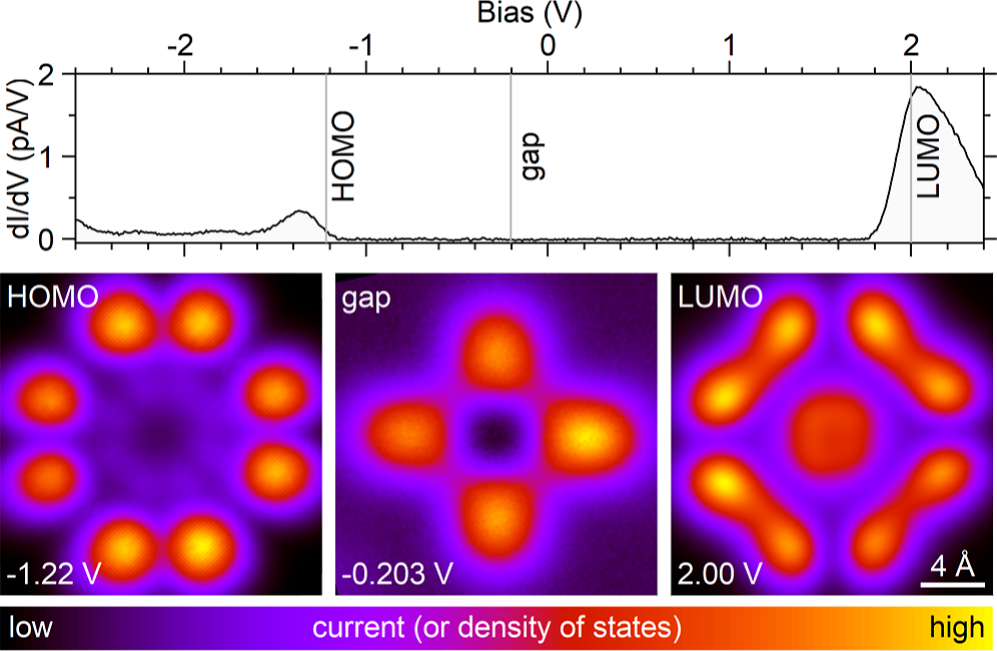
Typical differential conductance (d*I*/d*V*) spectrum on PtPc, indicating the energies of the molecular frontier orbitals with broad local maxima at −1.35 V (HOMO) and +2.05 V (LUMO). Bottom row: typical constant-height STM images of the frontier orbitals and of the feature appearing in the transport gap of the molecule. The color scale is valid for all figures with either measured (current) or calculated (DOS = density of states) images and spans the range from the minimum to the maximum of the absolute tunnel current. Here, the maxima of absolute currents are 66 pA (HOMO), 5.3 pA (gap feature), and 187 pA (LUMO). All images show an area of 20 × 20 Å^2^.

All experimental data shown here are measured in the constant-height mode (see Experimental Section methods for details). The data in Figure 2 are presented for comparison with the theoretical calculations discussed below. PtPc molecules are sensitive to the local inhomogeneity that results from the incommensurability of NaCl(100) with the herringbone reconstruction of Au(111). Consequently, some molecules are more stably adsorbed and may thus be preferentially selected by the experimentalist. Additionally, the substrate causes some molecules to adsorb with the 2-fold degeneracy of the LUMO lifted, enabling the imaging of one of the LUMOs in isolation. We chose tip-sample distances that enable imaging molecules with a good signal-to-noise ratio while maintaining stable scanning conditions. For excessively high currents and excessively short distances from the tip apex, molecules tend to move laterally, creating streaks in the image or even hop irreversibly to the tip.

The onset of this instability typically occurs at a tip-molecule distance of roughly 3 Å, accompanied by a maximum tunnel current of 200 pA, with substantial variability in both values. These values strongly depend on the applied voltage. In the experiments presented here, the concern is not only to minimize the distance to the molecule but also to maximize the image contrast of the radial components of the molecular features. The experimental images are intended to elucidate the effects of vacuum propagation in the electron tunneling process.

As we will demonstrate below, the density of states revealed in Figure 2 can be found within a survey of calculations for molecule-tip distances ranging from 4 to 8 Å. The figure shows measurements that represent typical measurements based on selected data. It is important to recall that we define the apex of the tip to be at *z* – *R*, where *R* is the curvature of the tip. Note also that the color bar used in Figure 2 is common to all 2D plots in this paper. Further details on its construction are given in the Supporting Information.

### Theoretical Results

**Figure 3:**
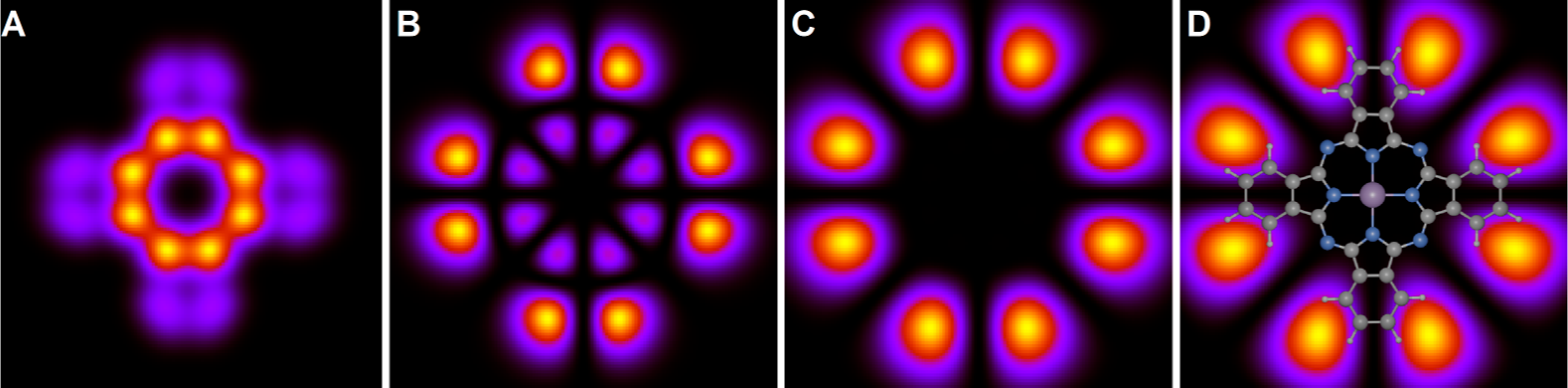
Calculated images at the energy of the HOMO (ε = −1.3 eV) for different distances from the molecular plane, *z* – *z*_0_ = 0 (A), 4 (B), 8 (C), and 12 Å (D). All panels show an area of 20 × 20 Å^2^. In panel D, the molecular structure is superposed.

We now present calculated images of a PtPc molecule. Figure 3 shows the results for the HOMO (ε = −1.3 eV) for different values of *z* – *z*_0_. The molecule has four “arms” along the *x*- and *y*-axes, with the HOMO featuring nodes along these axes, resulting in eight lobe-like features as a function of azimuthal angle. These lobes are particularly visible at larger *z* values. The HOMO image rapidly expands with increasing *z* and undergoes noticeable changes in its shape. As discussed in the Experimental Section above, some *z* values are too small to be experimentally accessible, yet observing the evolution with varying *z* yields valuable insights.

**Figure 4:**
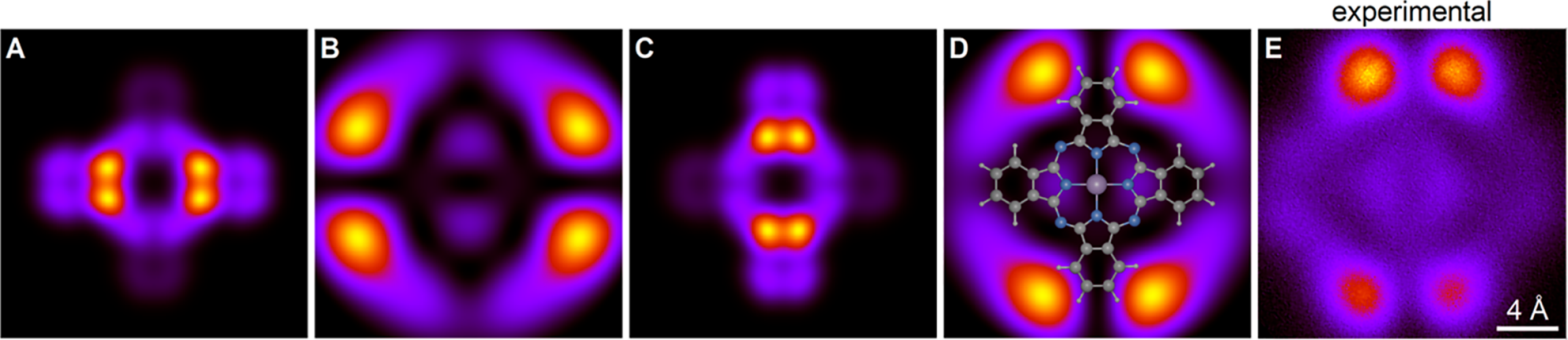
Calculated images of the two individual LUMO degeneracy lifted orbitals (ε = 1.7 eV) at the distances *z* – *z*_0_ = 0 Å (panels A and C) and 12 Å (panels B and D). All panels show an area of 20 × 20 Å^2^. In panel D, the molecular structure is superposed. Panel E shows a constant height STM image at a bias of only 1.35 V for a PtPc molecule for which the LUMO degeneracy happened to be lifted, probably due to a nearby substrate defect.

**Figure 5:**
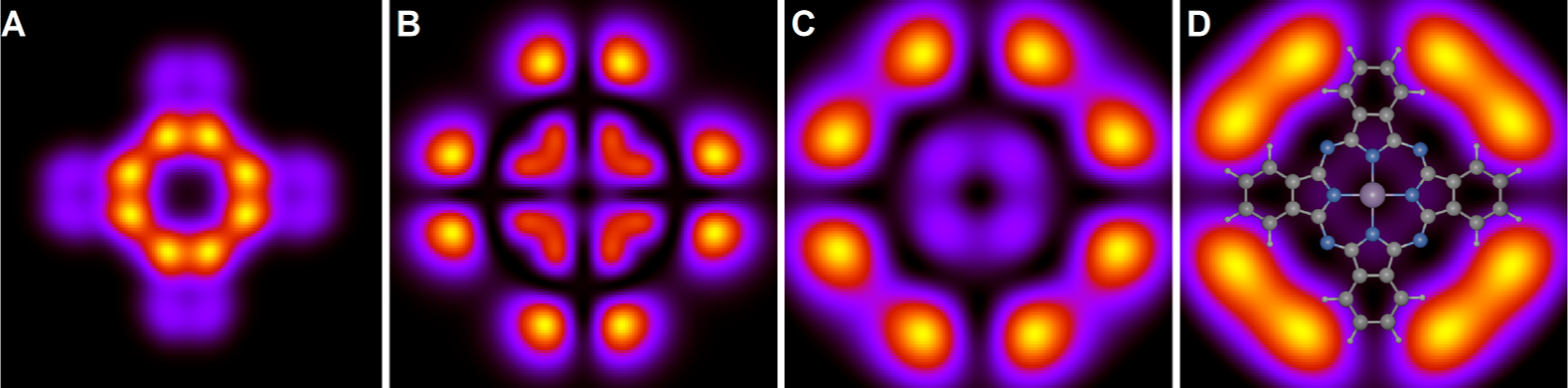
Calculated images of the sum of both LUMOs (ε = 1.7 eV) for distances, *z* – *z*_0_ of 0 (A), 4 (B), 8 (C), and 12 Å (D). All panels show an area of 20 × 20 Å^2^. In panel D, the molecular structure is superposed.

Figure 4 displays the results for the two degenerate LUMOs (ε = 1.7 eV) separately, while Figure 5 presents their sum, corresponding to the typical topographical image obtained by STM. One LUMO has most of its weight close to the *x*-axis, with a node along the *x*-axis. The other LUMO has its weight distributed analogously along the *y*-axis. The sum of the images results in eight features as a function of angle. Interestingly, for *z* – *z*_0_ = 0, the image bears a striking resemblance to the HOMO image at the same distance. As *z* increases, the LUMO image expands radially in a manner similar to the HOMO image.

The behavior of the angular features is quite different. Note the behavior near the high symmetry axes. For the HOMO, the eight angular features group into four pairs that straddle the positive and negative *x*- and *y*-axes. As *z* is increased, the features shift somewhat away from the *x*- and *y* axis, with the maxima moving toward, e.g., ±22.5° and minima at, e.g., ±45°. On the other hand, for the LUMO, this angular shift is much more important. Substantial weight is built up at, e.g., ±45°. Strikingly, this implies that weight accumulates in directions where the underlying molecule has no atoms.

**Figure 6:**
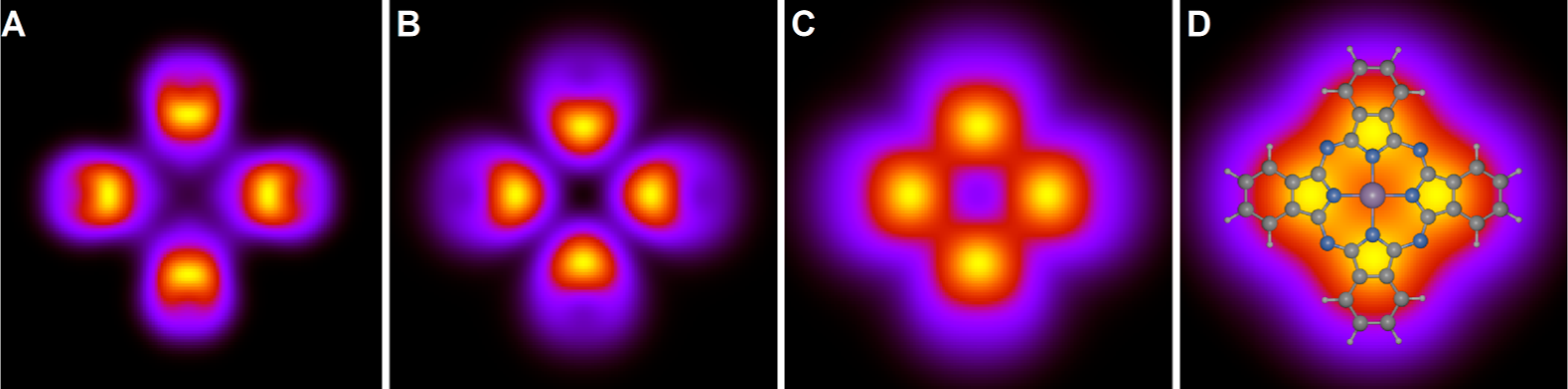
Calculated images of the states at the substrate Fermi energy (ε = 0.0 eV), that is in the molecular transport gap, for distances, *z* – *z*_0_ of 0 (A), 4 (B), 8 (C), and 12 Å (D). All panels show an area of 20 × 20 Å^2^. In panel D, the molecular structure is superposed.

One might have expected that for energies between the HOMO and LUMO, the image would show similarities to these MOs. However, even for energies very close to either the HOMO or LUMO, the images look qualitatively distinct from their corresponding orbitals. In contrast to the eight angular features of the HOMO and LUMO, only four features appear as a function of angle at voltages in the electronic transport gap (in-gap images). The HOMO image has no weight, and the image comprising the two degenerate LUMOs has little weight along the *x*- and *y*-axes. In stark contrast, in-gap images have substantial weight built up along these axes. Figure 6 shows that there is no clear radial expansion of the images with increasing *z*. This behavior stands in stark contrast to the clear expansion observed for the HOMO and LUMO.

Similar experimental results have been obtained for CuPc by Uhlmann et al.^57^ for the HOMO, LUMO, and energies in between. Our theoretical results for the HOMO and LUMO closely resemble the theoretical results obtained by Siegert et al.^45^ for CuPc at the similar tip-sample distance, and both agree with the experiment. Here, we discuss the reasons for the large deviations of these images from the shapes of the corresponding MOs inside the molecule. The theoretical results for energies lying between the HOMO and LUMO for CuPc, as reported in ref (44), significantly deviate from both our theoretical and experimental results for PtPc, as well as the experimental findings by Uhlmann et al. for CuPc.^57^ The reason is probably that the calculations in ref (44) did not include the lower-energy π-orbitals, which we demonstrate in the following to play a crucial role for topography images obtained at energies in the electronic transport gap of the molecule.

### Detailed Analysis of Electron Propagation Effects

We now turn our attention to the propagation of electrons through the vacuum region between the PtPc molecule and the tip. This is done by examining the intricate details of the coefficients that describe the electron wave function. In a forthcoming study, we will address the role of the propagation through the buffer (NaCl).

### Angular Distortions and Radial Expansion

We first discuss the exponential factor exp[−κ_*mi*_(ε)*z*], which describes the *z*-dependence in eqs 5 and 6 of the wave function amplitude. Table 1 presents the values of κ_*mi*_(ε). In parentheses, we show the relative damping of the intensity of components *mi*
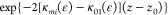
7relative to the *m* = 0 and *i* = 1 component for (*z* – *z*_0_) = 8 Å and ε = 0, close to the middle of the gap and well below the vacuum level at *V*_0_ = 4.3 eV. The table illustrates that (i) components for small *m* values are less damped and are thus strongly favored. For each *m* value, (ii) components with small values of *i* are strongly favored.

As discussed in the introduction, these effects emerge because a larger value of *m* or *i* leads to a larger *T*^∥^. Since we study electrons of a given energy ε, *T*_⊥_ is then correspondingly more negative and the decay in the *z*-direction exponentially more rapid.

**Figure 7:**
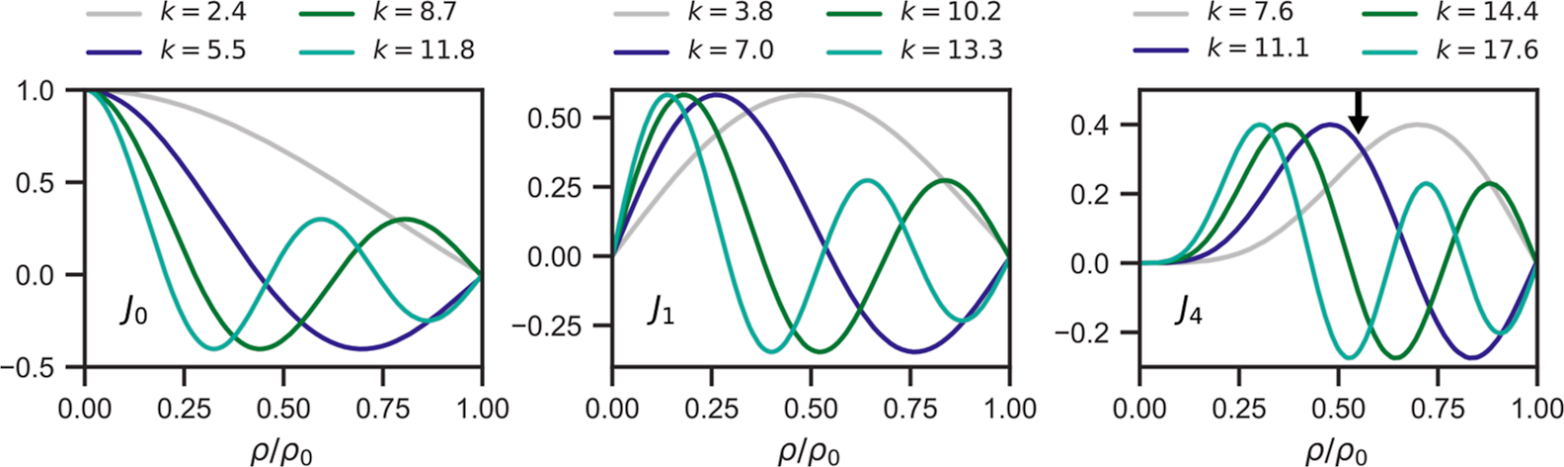
Bessel functions *J*_*m*_(*k*ρ/ρ_0_) for *m* = 0, 1, and 4 (from left to right) and for *k* = *k*_*mi*_, *i* = 1, 2, 3, and 4. The arrow in the right figure marks the positions of the outermost C atoms.

We now turn to the Bessel functions describing the radial behavior, that is the dependence on ρ. Figure 7 shows these functions for *m* = 0, 1, and 4. With exception of small values of *m*, e.g., *m* = 0 and *m* = 1, the function *J*_*m*_(*k*_*m*1_ρ) primarily characterizes the outer regions of the molecule, encompassing the outermost C atoms and the surrounding space outside these atoms. Conversely, the inner regions are predominantly characterized by *J*_*m*_(*k*_*mi*_) for *i* > 1.

Below we demonstrate that the two effects previously discussed, (i) and (ii), along with the corresponding behavior of the Bessel functions, are key for understanding the substantial distortion occurring in vacuum for the HOMO, LUMO, and the in-gap images.

### Highest Occupied Molecular Orbital

**Figure 8:**
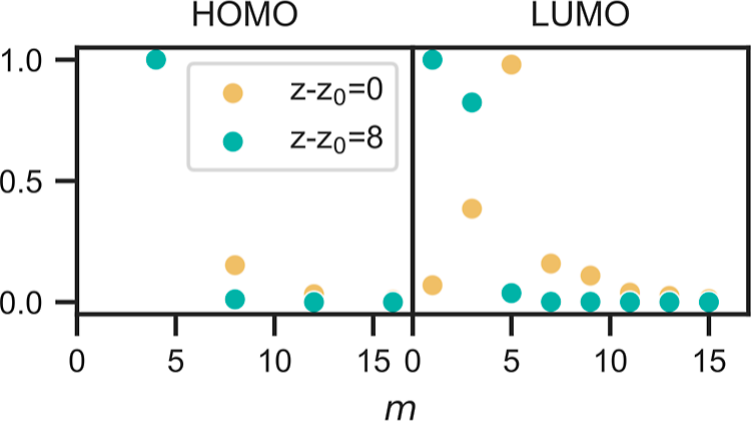
Relative weights of different *m* components in images of the HOMO (ε = −1.3 eV) and LUMO (ε = 1.7 eV) at distances *z* – *z*_0_ = 0 and 8 Å. For each value of *z*, the results are normalized to the maximum value for that *z*. For the HOMO, this is for *m* = 4, for both values of *z*, while for the LUMO, this is *m* = 5 for *z* – *z*_0_ = 0 and *m* = 1 for *z* – *z*_0_ = 8 Å.

We first discuss the HOMO in more detail. Given its eight features as a function of angle, it is described by angular functions with *m* = 4ν, ν = 1, 2, ···. The relative weight
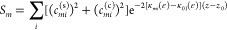
8is shown in Figure 8 as a function of *m* for ε = ε_HOMO_, highlighting the dominance of the *m* = 4 terms over higher values of *m* across all *z* values.

We first focus on *m* = 4. The numbers in parentheses in Table 1 show that the *i* = 1 component is significantly less suppressed than the components with *i* > 1 as *z* increases. This leads to a noticeable radial expansion of the image due to relatively higher weight of *J*_4_(*k*_4*i*_ρ) for *i* = 1 for large ρ than the *i* > 1 components (see Figure 7). Consequently, the centers of the eight HOMO lobes are positioned around *R* = 7.5 Å (0.63ρ_0_) for *z* – *z*_0_ = 8 Å, extending beyond the centers of the outermost C atoms at *R* = 6.6 Å (0.55ρ_0_) . Note that these contributions are not due to the H atoms and that suppressing the contributions from H atoms made no visible change in the image. Actually, the MOs with π character do not couple to the 1s H orbital, and σ orbitals, of importance in this context, have little 1s H character. The maximum of the image is at a somewhat smaller value of ρ than the maximum (ρ/ρ_0_ = 0.70) of *J*_4_(*k*_41_ρ/ρ_0_) due to the influence of mixed terms such as those with *i* = 1 and *i* = 2, even for *z* – *z*_0_ = 8 Å.

The *m* = 4 component results in eight intense features, prominently positioned at angles ±22.5° and separated by an angle of 45°. The higher order components, e.g., *m* = 8, move these features pairwise together toward the *x*- and *y*-axes, as is illustrated for the case of *z* = 1 Å. With increasing *z*, the influence of the *m* > 4 components diminishes rapidly, causing the angular placement of the features in each quadrant to approach ±22.5°. However, even for *z* – *z*_0_ = 8 Å, these features are weakly displaced toward the *x*- and *y*-axes due to the non-negligible contributions from higher *m*-values.

### Lowest Unoccupied Molecular Orbital

We now focus on the doubly degenerate LUMO. Figure 5 presents the sum of the images of both states. As mentioned earlier, HOMO and LUMO appear very similar for *z* – *z*_0_ = 0, both undergoing significant expansion in the ρ-direction with increasing *z*. However, their angular behavior diverges considerably. Notably, the summation of the LUMOs builds up intensity along the angles ±45 and ±135° for large values of *z*.

To understand this build up, we first notice that the LUMOs are described by terms sin(*m*ϕ) and cos(*m*ϕ) = sin(*m*ϕ + 90°) for odd values of *m*. Let us consider the one LUMO with prominent features close to the *x*-axis, described by sin(*m*ϕ) and shown in Figure 4A,B. The weights of its components with different *m*-values are shown in Figure 8. For *z* – *z*_0_ = 0, the *m* = 5 component has the most weight. This component has its maximum weight at the angle 90°/5 = 18°. As a result, this intensity appears approximately at ±18° for this LUMO, and naturally at 90 ± 18° for the other LUMO (Figure 4C,D). The *m* = 3 components are also relatively important, adding intensity that peaks at 90°/3 = 30°, thereby slightly shifting the overall intensity distribution away from the axes. The two arms of one LUMO (at ≈±18°) are then about 36° apart, while the two arms of two different LUMOs are about 90–36° = 54° apart. This leads to the features in Figure 5, displaying both LUMOs, being relatively close to the *x*- and *y*-axes for *z* – *z*_0_ = 0 Å.

We can now turn our attention to *z* – *z*_0_ = 8 Å. As demonstrated in Figure 8, the *m* = 1 and *m* = 3 components are the largest. For the LUMO with arms along the *x*-axis, these arms are shifted away from the axis, since sin(3ϕ) has its maximum for a larger ϕ compared to, e.g., sin(5ϕ), as evident in the right section of Figure 4 (for *z* – *z*_0_ = 12 Å). Similarly, for the other LUMO, the arms move away from the *y*-axis. The result is the buildup of an appreciable weight around, e.g., ϕ = 45°, even though there are no atoms in the underlying molecule in this direction.

### Gap States

Finally, we consider states in the electronic transport gap. These states were previously investigated in our earlier publication,^50^ wherein we concentrated on electron propagation from the substrate out to the PtPc molecule. However, in the current work, we focus on the propagation through the vacuum. A striking result in ref (50) was the rather simple image with four lobes along the *x*- and *y*-axes with no resemblance to the HOMO or LUMO, even though the measurement was performed for energies relatively close to the HOMO or LUMO. Furthermore, the state at the substrate Fermi energy (0.0 eV), Figure 6 illustrates that the weight of states within the transport gap of the molecule does not experience significant outward expansion. This is in contrast to the behavior observed in the case of the HOMO and the LUMO.

As discussed in ref (50), inside the PtPc molecule, the states within the molecule’s gap are to a good approximation linear combinations of bound PtPc MOs. Very close in energy to the HOMO, the HOMO dominates the gap state, producing a HOMO-like image (not shown here). However, moving up to energies above the HOMO, bound PtPc MOs below the HOMO and with small values of *m* rapidly begin to contribute significantly to the image for *z* = *z*_0_.

**Figure 9:**
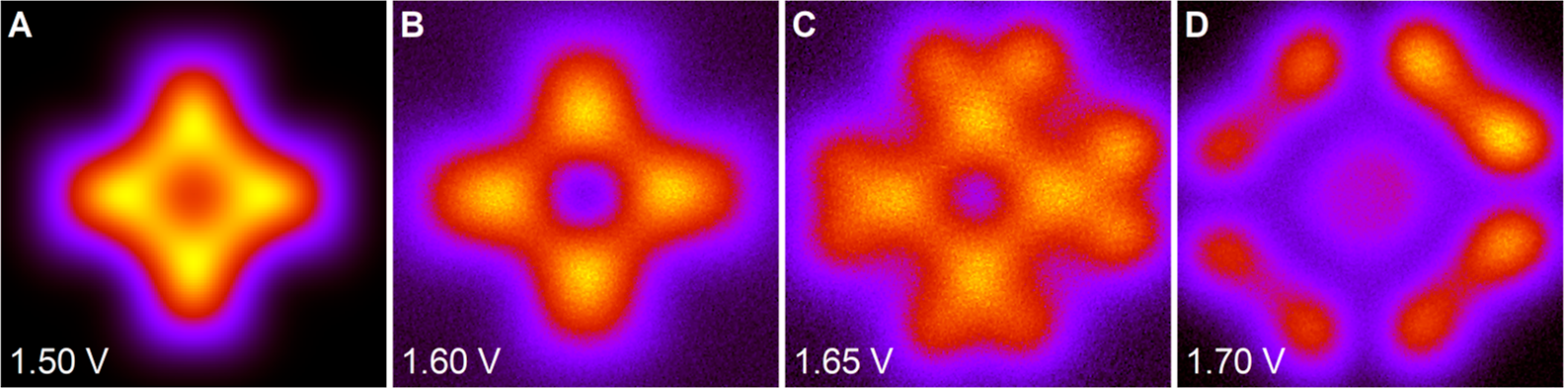
Constant height STM images (B–D) illustrating the sharp transition between the gap feature and the LUMO. The bias voltages are indicated in each panel. Panel A shows theoretical results for the bias 1.5 V. All panels show an area of 20 × 20 Å^2^.

We now discuss *z* > *z*_0_ considered in this paper. The smallest *m* value contributing to the HOMO is *m* = 4, as shown in Table 1, which efficiently undergoes exponential suppression as *z* is increased. This suppression then causes MOs with *m* = 0, 1, or 2 to gain relative prominence. The exponential suppression effect remains influential even for energies a few tenths of an eV above the HOMO, rendering components with smaller *m* values predominant in the image. Despite the corresponding molecular states being considerably lower in energy than the HOMO, these smaller *m* components dominate due to this strong exponential suppression with increasing *z*. Analogous phenomena occur at energies below the LUMO. This is illustrated by the sharp and reversible transition from imaging the gap feature and the LUMO within a few tens of meV as shown in the STM measurements in Figure 9B–D. The theoretical result (panel A) corroborates that this transition indeed happens very rapidly as a function of tunneling energy. Below, we show that the image is dominated by a π orbital 8 eV below *E*_F_. From the energy denominators in perturbation theory we may expect that for states at 0.1 V above the HOMO, the HOMO would contribute a factor (8/0.1)^2^ = 6400 more to the image than the lowest π orbital. However, the tunneling through vacuum favors the lowest π orbital exponentially over the HOMO. Together with effects in the buffer,^50^ this makes the lowest π orbital dominate over the HOMO. Given the great importance of orbitals below the HOMO and LUMO for images within the transport gap, it is not surprising that these images could not be described in ref (44), where these orbitals were not included.

We observe that the discussed effects render MOs below the HOMO very important for electron propagation at energies within the gap. However, there is an additional physical mechanism at play. Most of the MOs above the LUMO have many nodal surfaces since they have to be orthogonal to lower states. Consequently, these MOs then tend to have large *T*^∥^. For a given bias voltage, they tend to have a very negative *T*^⊥^, and therefore decay rapidly with increasing *z* values. This introduces a fundamental asymmetry between MOs well below versus most of those well above the gap.

For the HOMO and LUMO images, we found that the image strongly expands radially as *z* – *z*_0_ increases from 0 to 12 Å. By comparison with Figure 6, it becomes apparent that a similar strong expansion does not occur for the gap states. For the HOMO, the expansion was attributed to the increasing importance of the extended *i* = 1 states as *z* increases. A similar predominance of small values of *i* happens also for gap states. However, for these states, the *m* = 0 and *m* = 1 components are particularly important, and within this range of *m* values, the *i* = 1 and the *i* = 2 states have a comparable radial extension (see Figure 7) (note that if a much larger cylinder radius ρ_0_ is used, these arguments become more complicated since the basis states for small values of *i* are then very extended. However, the numerical results for the images, are essentially unchanged).

### Vacuum Propagation Strongly Favors Certain MOs

To gain a more comprehensive understanding of the origin of images for energies within the PtPc transport gap, we continue the PtPc MOs at specific energies ε into the vacuum region. The continuation, Ψ_ν_(ρ, ϕ, *z*, ε), for MO ν, is formulated in terms of the basis states employed in eq 5. The function Ψ_ν_ matches the MO ν continuously at *z* = *z*_0_. Next, we consider a solution  of the combined system comprising the substrate-buffer-PtPc and the vacuum at an energy ε_α_. Within the vacuum, we write the solution as
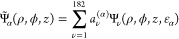
9

We then calculate the overlap integral, *S*_ν,μ_(*z*) of two functions Ψ_ν_ and Ψ_μ_, over a plane parallel to the surface and at a distance *z* from the molecule. Finally, we introduce a density matrix
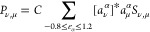
10where we sum over all states within the energy range from −0.8 to 1.2 eV in the transport gap. To ensure normalization, the constant *C* is chosen so that
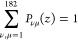
11

It is notable that some of the off-diagonal terms might be negative. This matrix illustrates how the propagation through the different MOs contributes to the image.

**Figure 10:**
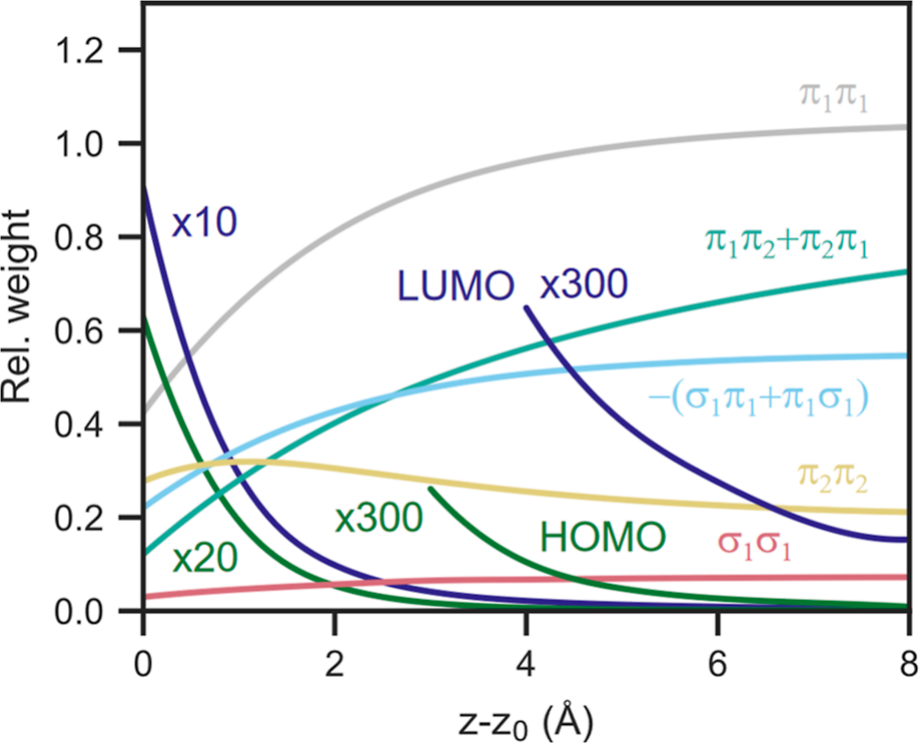
Theory predicted weights *P*_ν,μ_(*z*) describing contributions to the image due to products of tails of important MOs. The product was integrated over a plane at *z* – *z*_0_ and over the bias range (−0.8 eV ≤ ε ≤ 1.2 eV). We show diagonal elements, *P*_νν_, for the lowest π-orbital (π_1_, ε – ε_F_ = −8 eV), a π-orbital with an approximately cylindrical nodal surface (π_2_, ε – ε_F_ = −5 eV), the diagonal terms summed over seven orbitals about 1.8 eV below the HOMO as well as the HOMO and LUMO. The latter are plotted twice, each with the indicated enhancement factor. We also show two off-diagonal contributions involving the lowest σ orbital (σ_1_, ε – ε_F_ = −26 eV) and π_1_-orbital as well as the π_1_ and π_2_ orbitals.

**Figure 11:**
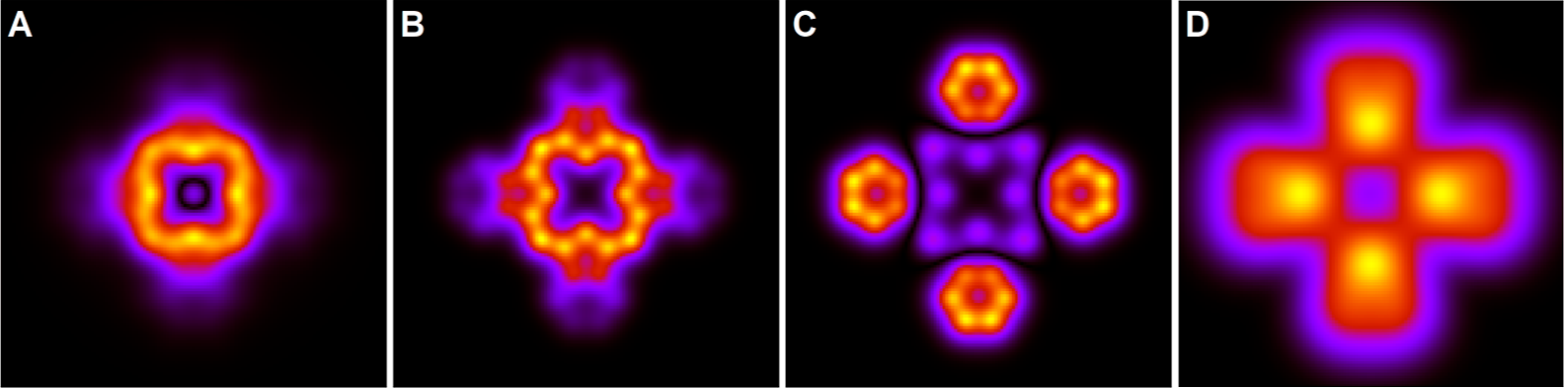
Important MOs for images in the gap. The panels show the absolute value of (A) the lowest σ MO (σ_1_ in Figure 10), (B) the lowest π MO (π_1_), and (C) a π MO with one “radial” node (π_2_). The second MO (π_1_) is positive everywhere (for *z* > 0), while the first MO (σ_1_) is slightly negative at parts very close to the Pt nucleus (≤0.7 Å) due to the Pt 3*z*^2^ – *r*^2^ d-orbital. We emphasize the similarity of the σ_1_ and π_1_ orbitals. The inner parts of π_2_ are negative and the outer parts are positive. In constructing the images from Figures 3–6 the coefficient of the σ orbital has the opposite sign to the coefficients of the two π-orbitals which leads to an overall cancellation of weight in the inner parts of the image. The orbitals were calculated at *z* – *z*_0_ = 0 Å. Panel D shows the calculated image at −1.0 V (a value just above the HOMO energy at −1.3 V) for *z* – *z*_0_ = 6 Å. All panels show an area of 20 × 20 Å^2^.

Figure 10 shows the *z*-dependence of important elements of *P*_νμ_. Some of the corresponding orbitals are shown in Figure 11. Even the lowest (σ_1_) state, at −26 eV, contributes more significantly than the HOMO (−1.3 eV) and the LUMO (1.7 eV). As previously discussed, this phenomenon stems from the lowest σ orbital having only a small positive *T*^∥^, due to the absence of nodes in this plane. For a given total energy ε, *T*^⊥^ is then not very negative, and the exponential decay in vacuum not so rapid.

Similar effects happen for the lowest π orbital, π_1_, although its smaller energy difference to ε_F_ = 0 results in a significantly larger contribution. The π_2_ orbital has a radial node (as seen in Figure 11), resulting in a somewhat larger *T*^∥^, a more negative *T*^⊥^, and consequently, a more rapid decay in the vacuum. Panel D shows the calculated image at a value just above the HOMO energy. It indeed shows great similarities with a linear combination of the orbitals in panels B and C.

The HOMO has components with *m* = 4 or higher *m* values. Table 1 shows that the *m* = 4 components decay rapidly. As shown in the Supporting Information, most of the HOMO weight is furthermore in components with fairly large values of *i* ∼ 4. This configuration results in *T*^∥^ being very large, and a corresponding rapid decay outside the molecule. We have also summed the diagonal contributions from 7 orbitals (“7 orbitals”), within a calculated energy range of approximately 0.1 eV, and around 1.8 eV below the HOMO. These orbitals have much more weight than the HOMO and the LUMO, rendering them potentially important in some specific contexts. The Supporting Information shows that the leading components of the LUMO are also characterized by large values of *m* and *i*, and the corresponding rapid decay in vacuum. However, the LUMO also has components with small values of *m* and *i*. Despite these components’ small amplitudes at the matching surface (*z* – *z*_0_ = 1 Å), their relatively slow decay in vacuum results in their significant contribution. Furthermore, much of the contribution from the LUMO comes from the upper part of the studied energy interval, where the wave functions generally decay more slowly. Consequently, the LUMO contribution (4 × 10^–4^ from both LUMOs) is not as drastically reduced during the propagation through vacuum as observed for the HOMO contribution (2 × 10^–5^). The observation that the most important contributions stem from orbitals lacking angular nodes (as shown in Figure 10) elucidates the shapes of the images presented in Figure 6.

## Conclusions

We have analyzed the transport of electrons through vacuum in STM studies of PtPc adsorbed on a thin NaCl film atop an Au substrate. For vacuum propagation, basis states with few nodes in the angular and radial directions parallel to the surface are exponentially favored. Their weak variations parallel to the surface result in a small positive contribution, *T*^∥^, to the kinetic energy. For a given bias, such basis states, by construction, then have an only moderately negative contribution, *T*^⊥^, to the kinetic energy from variations perpendicular to the surface. This corresponds to a moderate exponential decay perpendicular to the surface. These basis states therefore dominate the image for large tip distances. When considering the HOMO or LUMO, both experimental and theoretical analyses reveal a substantial radial expansion of the image. This expansion is attributable to the dominance of vacuum basis states that lack radial nodes. In the case of the LUMO, there is also a substantial angular distortion, due to the additional emphasis of basis states with few angular nodes. This leads to a substantial weight along the diagonals *x* = *y* and *x* = −*y*, despite the underlying molecule having no atoms at these positions. The analysis moreover demonstrates that the low resolution observed for orbitals with many nodes also has its physical origin in the electron propagation into vacuum and is not exclusively imposed by the limited spatial resolution of the STM tip. For propagation within the transport gap, the image is due to a linear combination of waves originating from many MOs. Notably, the significance of HOMO and LUMO quickly reduces when entering the transport gap, already at energies just a few tenths of an eV above the HOMO or below the LUMO energy, leading to a radical change in the resulting STM topography image. Waves characterized by few angular and radial nodes then make a large contribution. Particularly, this accentuates the role of π-MOs located energetically well below the HOMO. These intricate details provide a consistent and rational basis for understanding the investigative capabilities of the STM.

## Experimental Section

The experiments were performed in a home-built low-temperature (*T* ∼ 5 K) STM operated under ultrahigh vacuum at a base pressure of ≤1 × 10^–11^ mbar.^58^ The preparation of single molecules of PtPc adsorbed on three monolayer NaCl(100) on Au(111) follows a procedure described previously.^49,50^

## Data Availability

The data supporting this study’s findings are available from the corresponding authors upon reasonable request.

## Supporting Information Available

TB model—NaCl(100) on Au(111), TB model—PtPc, propagation in vacuum, and color bar generation (PDF)
