# Supplementary material for: Scanning Tunneling Microscopy for Molecules: Effects of Electron Propagation into Vacuum
Source: ACS Nano. 2024 Apr 29;18(19):12158–67. doi: 10.1021/acsnano.3c12315 (PMC11100283; doi:10.1021/acsnano.3c12315)
Supplement: Supplementary file 1 — nn3c12315_si_001.pdf [file nn3c12315_si_001.pdf]

Supplementary Information for Scanning Tunneling Microscopy for Molecules: Effects of Electron Propagation into Vacuum

Abhishek Grewal,[†] Christopher C. Leon,[†] Klaus Kuhnke,[*,†] Klaus Kern,[†,‡] and Olle Gunnarsson[*,†]

†*Max-Planck-Institut für Festkörperforschung, Heisenbergstraße 1, 70569 Stuttgart, Germany*

‡*Institut de Physique, École Polytechnique Fédérale de Lausanne, Lausanne, 1015, Switzerland*

E-mail: k.kuhnke@fkf.mpg.de; o.gunnarsson@fkf.mpg.de

Tight-binding model – NaCl(100) on Au(111)

We use the same model for NaCl on Au as in our earlier work.[1] This model consists of a NaCl(100) three-layer film on a Au(111) substrate. The NaCl film contains $9 \times 9 \times 4$ atoms per layer. We use three different clusters representing the Au substrate and average the results. The three Au clusters have four, six, or eight layers with 1020, 780, and 572 Au atoms per layer, respectively. In total there are then 486 Na, 486 Cl, and 4080, 4680, or 4576 Au atoms. We impose periodic boundary conditions parallel to the surface for both, the NaCl slab and the Au slabs. Hopping integrals are constructed according to the nearest neighbor hopping rules rules of Harrison,[2,3] including $s - d$[3] and $p - d$ hopping. The NaCl and Au surfaces are non commensurate. We place the central Na atom on top of a central Au atom in the neighboring NaCl and Au layers.

To describe the Au substrate, we use the lattice parameter $a_{\mathrm{Au}} = 4.07$ Å.[4] We use the Harrison level energies $\varepsilon_{6s} = -6.98$ eV and $\varepsilon_{5d} = -17.78$ eV as a starting point and add a $6p$ level at 5 eV above the $4s$-level. We then shift the $5d$-level relative to the $6s$ and $6p$ levels so that the top of the $5d$ band is placed at 1.7 eV below the Fermi energy.[5] Finally all energies are shifted so that the Fermi energy is at zero. The resulting parameters are summarized in Table 1.

To describe the NaCl film, we essentially follow our earlier work[1,6] and choose parameters such that the conduction band has mainly Cl $4s$ character.[6,7] For this purpose we replace the Cl $3s$ level by a $4s$ level, which has been strongly lowered by the Madelung potential, while the Na levels are shifted strongly upwards. We adjust the Harrison parameters so that the experimental band gap (8.5 eV)[8] is reproduced for bulk NaCl. Image potential effects were neglected. According to calculations[9] using the GW method,[10] the top of the valence band is 5 eV below the Fermi energy.

Table 1: Level energies used for PtPc, NaCl and Au after shifts described in the text. The Fermi energy is put at zero.

Element	s	p	d
Au (6s, 6p, 5d)	4.1	9.1	-3.7
Na (3s, 3p)	12.8	16.8	-
Cl (4s, 3p)	10.2	-5.0	-
C (2s, 2p)	-19.38	-11.07	-
N (2s, 2p)	-26.22	-13.84	-
Pt (6s, 5d)	-6.85	-	-16.47
H (1s)	-13.61	-	-

We then shift all the NaCl energies relative to the Au energies correspondingly. The resulting parameters are shown in Table 1. The calculations were performed using the lattice parameter $a_{\mathrm{NaCl}} = 5.54$ Å.[11]

We use the calculated separation $d_{\mathrm{Au-NaCl}} = 3.12$ Å between the Au surface and the NaCl film.[11] Since the NaCl film and the substrate are incommensurate, several Au atoms can have similar distances to a given NaCl atom, and the nearest neighbors are poorly defined. We then use a smooth distance dependent cut off of the Harrison prescription for the hopping between the substrate and the film. Thus the Harrison prescription for these hopping integrals is multiplied by a factor

$$\exp^{-(d-d_{\mathrm{Au-NaCl}})^2/\lambda_{\mathrm{SB}}^2}, \tag{1}$$

where d is the distance between an Au atom and a NaCl atom at the Au-NaCl interface. Here λ_{SB} is chosen such that summing these factors over all the Au neighbors of a NaCl atom and averaged over the NaCl atoms in the innermost layer adds up to four. Then the innermost NaCl atoms effectively couple to four Au atoms.

Tight-binding model – PtPc

We study the absorbed molecule platinum phthalocyanine (PtPc). The coordinates are obtained from a density functional calculation. The tight-binding parameters are primarily obtained from Harrison.[2] For the H atoms we include the 1*s* level at the energy -13.6 eV (not given by Harrison). Guided by Miwa *et al.*[12] we use the separation 3.4 Å between the the PtPc molecule and the NaCl film. PtPc is absorbed atop a Na atom. For PtPc the four "arms"of the molecule are along the NaCl (100) directions. For the hopping between the molecule and NaCl, we also use the rules of Harrison,[2] but modified as in Eq. (1) above. Again a λ_{BM} is chosen so that, on average, from each atom in the molecule there is effectively hopping to four atoms in the NaCl buffer. The Au slab breaks the four-fold symmetry of PtPc which has been reintroduced in the plots.

These parameters incorrectly puts a σ-orbital below the HOMO. We therefore shift this orbital by 3.2 eV upwards, slightly above the LUMO. We also adjust the parameters so that the experimental gap is obtained, including image effects. Finally we align the levels with the Fermi energy

($E_F = 0$) of the system, so that the HOMO is located at -1.3 eV and LUMO at 1.7 eV, in agreement with experiment. The resulting parameters are shown in Table 1.

Propagation in vacuum

In the main text [Eq. (4)], we presented the expansion of the MOs of PtPc in basis function appropriate for vacuum propagation

$$\sum_{mi}[c^{(s)}_{mi}\sin(m\phi) + c^{(c)}_{mi}\cos(m\phi)]J_m[k_{mi}\rho]e^{-\kappa_{mi}z}, \tag{2}$$

where $m(\geq 0)$ is an integer and J_m is an integer Bessel function. The values of κ_{mi}, determining the exponential decay with z, were given in Table I in the main text. As discussed in that context, the different components mi decay at very different rates in vacuum, with the $m = 0$ and $i = 1$ component decaying most slowly. Table 2 shows the expansion coefficients for some important MOs in terms of these vacuum functions. The largest component for the lowest σ and lowest π orbitals is the $m = 0$ $i = 1$ component, which therefore decay relatively slowly with z. The π orbital with one radial node has somewhat larger amplitudes for higher components, and decays somewhat faster. The HOMO only has components with $m = 4$ or higher m values, which decay rapidly with z. In particular, most of these components, in addition, have rather high values of i, making the decay even faster. The LUMO has the largest amplitude for an $m = 5$ component with a high i value. This component decays very rapidly. However, there are also components

Table 2: Largest expansion coefficients for a few MOs [Eq. (2)]. In addition, we show a few coefficients for small i and m values. The table shows results for the lowest σ MO, the lowest π MO, a π MO with one radial node as well as the HOMO and one LUMO. For a given orbital, the coefficients have been renormalized so that the largest coefficient is unity.

Lowest σ			Lowest π			Higher π			HOMO			LUMO		
m	i	$c^{(c)}_{mi}$	m	i	$c^{(c)}_{mi}$	m	i	$c^{(c)}_{mi}$	m	i	$c^{(s)}_{mi}$	m	i	$c^{(c)}_{mi}$
0	1	1.00	0	1	1.00	0	1	0.81	4	1	-0.23	1	1	-0.03
0	2	0.78	0	2	0.73	0	3	-0.92	4	3	0.41	1	2	0.13
0	4	-0.32	0	4	-0.36	0	5	0.47	4	4	1.00	3	4	-0.69
0	5	-0.46	0	5	-0.40	4	1	0.62	4	5	0.90	5	4	1.00
0	6	-0.36	0	6	-0.25	4	2	1.00	4	6	0.42	5	5	0.97
0	7	-0.16	4	5	0.17	4	3	0.47	8	3	-0.38	5	6	0.56

with $m = 1$ and $m = 3$, which have small amplitudes, but are still important because of their slower decay. Overall, the LUMO therefore does not decay as rapidly as the HOMO.

Color bar generation

Let $\gamma = 1.25$ and the range of x be $[0, 1]$. The color bars of the main text are parameterized by the following equations for each primary color component.

$$\text{red}(x) = x^{\gamma/2} \tag{3}$$

$$\text{green}(x) = x^{3\gamma} \tag{4}$$

$$\text{blue}(x) = \begin{cases} \sin(2\pi(x^{\gamma})) & 0 \leq x^{\gamma} \leq 0.5 \\ 0 & 0.5 < x^{\gamma} \leq 1 \end{cases} \tag{5}$$

References

1. Grewal, A.; Leon, C. C.; Kuhnke, K.; Kern, K.; Gunnarsson, O. Character of Electronic States in the Transport Gap of Molecules on Surfaces. *ACS Nano* **2023**, *17*, 13176–13184.

2. Harrison, W. *Elementary Electronic Structure*; WORLD SCIENTIFIC, 1999.

3. Harrison, W. *Electronic Structure and the Properties of Solids: The Physics of the Chemical Bond*; Freeman, 1980.

4. Davey, W. P. Precision Measurements of the Lattice Constants of Twelve Common Metals. *Phys. Rev.* **1925**, *25*, 753–761.

5. Sheverdyaeva, P. M.; Requist, R.; Moras, P.; Mahatha, S. K.; Papagno, M.; Ferrari, L.; Tosatti, E.; Carbone, C. Energy-Momentum Mapping of d -Derived Au(111) States in a Thin Film. *Phys. Rev. B* **2016**, *93*, 035113.

6. Leon, C. C.; Grewal, A.; Kuhnke, K.; Kern, K.; Gunnarsson, O. Anionic Character of the Conduction Band of Sodium Chloride. *Nat. Commun.* **2022**, *13*, 981.

7. de Boer, P. K.; de Groot, R. A. The Origin of the Conduction Band in Table Salt. *Am. J. Phys.* **1999**, *67*, 443–445.

8. Poole, R. T.; Jenkin, J. G.; Liesegang, J.; Leckey, R. C. G. Electronic Band Structure of the Alkali Halides. I. Experimental Parameters. *Phys. Rev. B* **1975**, *11*, 5179–5189.

9. Wang, S.; Kharche, N.; Costa Girão, E.; Feng, X.; Müllen, K.; Meunier, V.; Fasel, R.; Ruffieux, P. Quantum Dots in Graphene Nanoribbons. *Nano Lett.* **2017**, *17*, 4277–4283.

10. Hedin, L. New Method for Calculating the One-Particle Green's Function with Application to the Electron-Gas Problem. *Phys. Rev.* **1965**, *139*, A796–A823.

11. Chen, H.-Y. T.; Pacchioni, G. Properties of Two-Dimensional Insulators: A DFT Study of Co Adsorption on NaCl and MgO Ultrathin Films. *Phys. Chem. Chem. Phys.* **2014**, *16*, 21838–21845.

12. Miwa, K.; Imada, H.; Kawahara, S.; Kim, Y. Effects of Molecule-Insulator Interaction on Geometric Property of a Single Phthalocyanine Molecule Adsorbed on an Ultrathin NaCl Film. *Phys. Rev. B* **2016**, *93*, 165419.
